# Management of oral aphthous ulcer: A review

**DOI:** 10.6026/973206300200434

**Published:** 2024-05-31

**Authors:** Bhart Vashishat, Shruti Sinha, Tanmay Srivastava, Anuj Mishra, Kavleen Kaur Sethi, Sparsh Srivastava, Pratik Surana

**Affiliations:** 1 Department of Oral and Maxillofacial Surgery, Institute of Dental Sciences, Bareilly, Uttar Pradesh, India; 2 Department of Oral Medicine and Radiology, Saraswati Dental College and Hospital, Lucknow, Uttar Pradesh, India; 3 Department of Oral Medicine and Radiology, Sardar Patel Post Graduate Institute of Dental and Medical Sciences, Lucknow, Uttar Pradesh, India; 4 Department of Orthodontics and Dentofacial Orthopaedics, BBD College of Dental Sciences, Lucknow, Uttar Pradesh, India; 5 Department of Pedodontics and Preventive Dentistry, Maitri College of Dentistry and Research Centre, Durg, Chhattisgarh, India

**Keywords:** Aphthous ulcers, Pain, Management

## Abstract

Aphthous ulcers, also known as canker sores, are a common oral condition characterized by recurrent, painful, small ulcers that typically arise on the non-keratinized mucous membranes within the mouth. Although the pathogenesis of aphthous ulcers is not completely understood; it is believed to be involved in a combination of genetic predisposition, local trauma, stress, hormonal changes, and certain environmental factors. Thus, management of aphthous ulcer revolves around reducing pain, promoting healing and preventing recurrence.

## Background:

Aphthous ulcers or canker sores are one of the most prevalent oral mucosal conditions affecting a significant percentage of the population. [1] These lesions can cause considerable discomfort, impacting eating, speaking, and quality of life. [2] The exact etiology remains unclear, but several factors have been associated with the development and exacerbation of aphthous ulcers such as minor mechanical injuries to systemic conditions such as nutritional deficiencies, celiac disease, inflammatory bowel diseases, and Behçet's disease. [3] Clinically, aphthous ulcers present as round or ovoid, small, and shallow lesions with a white or yellow fibrous base surrounded by an erythematous halo. [4] The diagnosis is typically clinical, based on the patient's history and the appearance of the lesions. [5] Aphthous ulcers are categorized into minor, major, and herpetiform ulcers, with minor ones being the most common. [6] Management strategies can broadly be classified into palliative treatments, aimed at alleviating pain, and active treatments, which are directed at healing and preventing future occurrences. The therapeutic approach is multifaceted, including topical agents for symptomatic relief, systemic medications for more severe or refractory cases, and natural and alternative remedies that have been explored for their benefits in treating and preventing aphthous ulcers. [1,2]

## Etiopathogensis:

The exact etiopathogenesis of aphthous ulcers is not completely understood; however, several contributing factors have been identified. [3]

## Genetic predisposition:

There seems to be a hereditary component to aphthous ulcers, with many patients reporting a family history of the condition. [7]

## Immune system response:

Abnormal immune system reactions, where the body's defense mechanism attacks and destroys healthy mouth cells, could be responsible. This might be related to a T-cell mediated immune response. [4]

## Hormonal fluctuations:

Changes in hormones, particularly during menstrual cycles, may precipitate the formation of canker sores in some women. [3]

## Nutritional deficiencies:

Deficiencies in certain vitamins and minerals, such as B12, iron, and folic acid, have been linked to an increased likelihood of developing aphthous ulcers. [3]

## Microbial influences:

While no infectious agent has been consistently linked to canker sores, an imbalance of the normal oral microbiota may be a contributing factor. [2]

## Mucosal injury:

Minor injuries to the mouth from dental work, accidental biting, or aggressive tooth brushing can lead to the development of these ulcers. [4]

## Stress:

Emotional stress is often cited by patients as a precipitating factor for aphthous ulcer outbreaks. [5]

## Gastrointestinal diseases:

Conditions such as Celiac disease, inflammatory bowel diseases like Crohn's and ulcerative colitis, and Behçet's disease are sometimes associated with the occurrence of aphthous ulcers. [6]

## HIV/AIDS:

Aphthous-like ulcers are often more severe and may be more frequent in individuals with HIV/AIDS, suggesting immunodeficiency can be a contributing factor. [2, 6]

## Clinical features:

Aphthous ulcers are small, painful lesions that typically develop on the movable parts of the mouth's mucous membrane, such as the inner surfaces of the lips, cheeks, tongue, and soft palate. [7] Clinically, they present as oval or round sores with a white or yellowish fibrinous center, circled by a halo of erythematous (red) mucosa. [8] Their size can vary from a few millimeters to over a centimeter. Aphthous ulcers are categorized into minor, major, and herpetiform types, with minor being the most common. These lesions often cause significant discomfort, especially when eating, speaking, or brushing teeth. [9] The development of an aphthous ulcer typically proceeds through an initial prodromal stage comprising tingling or a burning sensation before the sore actually appears. The ulcer then manifests as a round or ovoid lesion with a yellowish-white fibrinoid center and a red haloes. The healing occurs spontaneously within 1-2 weeks without scarring. [8]

## Management of aphthous ulcer:

Management is generally aimed at symptom relief (such as topical anesthetics), promoting healing (with topical corticosteroids or antimicrobial mouthwashes), and maintaining good oral hygiene; some individuals with severe, recurrent sores, further investigation to rule out systemic conditions may be warranted. [10,11, 12]

## Dietary modification:

Regarding the role of nutrition in treating aphthous ulcers, there is no data available. [13] Avoiding substances that most patients report regularly causing ulcers is advised, particularly if the patient has observed a correlation. In general, one should stay away from hard, acidic, and salty foods and drinks, including citrus fruits, tomatoes, fruit juices, and spices like curry, paprika, and pepper. It's also preferable to stay away from dental care products that contain sodium lauryl sulphate (SLS). Using toothpaste without SLS shortened the time it took for oral aphthous ulcers to heal and decreased their pain. [14,15]

## Topical therapies:

Topical therapies and specialized mouth rinses play a pivotal role in managing a variety of oral health conditions due to their targeted approach. For instance, topical analgesics like lidocaine and benzocaine offer substantial pain relief by numbing the mouth's sensitive areas, typically in response to ulcers or the aftermath of dental work. [11] Equally important are topical steroids such as triamcinolone acetonide and fluocinonide, which combat inflammation robustly, facilitating the healing of inflamed or nascent lesions. [12] They're usually formulated into adhesive pastes that stay in place post-application, ideally after meals and just before sleep, to maximize their therapeutic effect. [16] In situations where a wound or ulcer is exposed to potential irritants, substances like cyanoacrylates provide a welcome shield. They create a protective layer over the area to fend off aggravation from food or dental apparatus which can enhance healing but may need frequent reapplication due to the natural movements and functions of the mouth. [16] Furthermore, antiseptic agents containing various tinctures aid in disinfecting lesions and curtail the threat of subsequent infections. They work by breaking apart the cellular walls of harmful microorganisms and should be concentrated enough to be effective while maintaining a safe profile to prevent tissue damage or discomfort. [12] Switching the focus to mouth rinses, antimicrobial mouthwashes that contain chlorhexidine gluconate are prominent for their ability to subdue bacterial colonies within the mouth. The benefits are clear; these rinses thwart additional infections in oral lesions and are adept at staving off gingivitis however, one must be wary of possible side effects, which can include alterations in taste sensation and teeth staining with long-term use. [14] Antibiotic mouthwash contains tetracycline; due to its capacity to inhibit collagenase activity, it lessens the size, length, and discomfort of ulcers. [17] Oral ulcer pain has been observed to be temporarily relieved by benzodamine mouthwash, but healing is not aided by this treatment. [18]

## Systemic therapy:

When oral health conditions escalate to severe levels or are accompanied by persistent and intense pain, as seen with major aphthous ulcers, systemic medications can play a vital role in management. One common systemic treatment option is oral prednisone, a potent corticosteroid that can substantially reduce inflammation and immune response. [15] Administering prednisone often brings about rapid relief from pain and swelling; however, its use must be carefully managed due to the potential for significant side effects, particularly with long-term use, such as increased susceptibility to infection, osteoporosis, and altered glucose metabolism. [19] Beyond these, other immuno-modulatory agents such as colchicine, dapsone, or azathioprine have been employed in the management of severe oral health conditions. [17] Colchicine, traditionally used for gout, has anti-inflammatory properties; dapsone, an antibacterial, is also known to suppress the immune system; and azathioprine, another powerful immunosuppressant, is commonly used in organ transplant recipients. These medications can offer significant benefits but are not without risks. Their potential to cause systemic toxicity necessitates regular and close monitoring of patients, typically involving periodic blood tests to assess the impact on the liver, kidneys, and hematologic system and ensure the continued safety of the therapy. [20,21] Most individuals with chronic recurrent oral pharyngitis respond well to colchicine (0.5-2 mg daily). Depending on the severity of the ulcers and how well the medicine is tolerated, an off-label trial with 1-2 mg daily is advised for a duration of 6 weeks, after which long-term therapy may be necessary. [23,23] Colchicine shown a discernible improvement in 63% of cases over a three-month period and in 37% over several years in a large open research conducted by Fontes *et al.* Of the patients, 41% had at least a 50% reduction in the number and duration of aphthous ulcers, while 22% were disease-free. [24] Aphthous ulcers can become painful and widespread in people with advanced HIV infection or impaired immune systems. According to certain research, thalidomide may facilitate the healing process for oral aphthous ulcers. Out of the 29 patients in the thalidomide group, 26 (90 percent) had complete or partial responses at the end of week 4, according to a double-blinded trial on the use of thalidomide as therapy for oral aphthous ulcers in HIV-positive individuals. [25,26] Thalidomide is useful in the treatment of oral aphthous ulcers; however, due to its toxicity, adverse effects, and high cost, it should only be utilised in situations where oral corticosteroids are not an option. [27]

## Recent advances in management strategies:

Recent advances in managing aphthous ulcers, also known as canker sores, have encompassed a range of novel approaches, including: [15]

## Biologics:

Medications that target specific components of the immune system have shown promise. For instance, drugs like TNF-alpha inhibitors, which are commonly used in autoimmune disorders like rheumatoid arthritis, are being explored for recalcitrant cases of aphthous ulcers. [9, 10]

## Laser therapy:

Low-level laser therapy has been reported to reduce pain and promote faster healing of aphthous ulcers by reducing inflammation and modulating pain signals. [15] It is believed that wounds can trigger re-epithelization when exposed to low power lasers. Several hypotheses have been put forth to clarify how they work. Low-power lasers have been proposed to improve reepithelization through raising respiratory metabolism, which in turn raises collagen production, mitotic activity, and epithelial growth. [28]

## Natural and alternative remedies:

There is increasing interest in the use of natural and alternative remedies. [29] Herbal preparations, essential oils, and other natural products are being scientifically evaluated for efficacy and safety. [30, 31]

## Stress management:

Stress is a well-documented trigger for aphthous ulcers. Techniques for stress reduction, including cognitive-behavioural therapy, mindfulness, and biofeedback, may have a role in comprehensive management plans. [31, 32]

## Conclusion:

The management of aphthous ulcers primarily focuses on alleviating pain, hastening healing and reducing recurrences. It involves a combination of topical treatments like corticosteroids and antiseptics, systemic medications for severe cases, and lifestyle adjustments to mitigate potential triggering factors. Addressing nutritional deficiencies and stress may also benefit prevention. While these ulcers are generally self-limiting, effective management is key to improving quality of life for affected individuals.
